# Penoscrotal edema: a case report and literature review

**DOI:** 10.1186/s12894-019-0456-6

**Published:** 2019-04-15

**Authors:** Tian Lin, Yun-Zhi Lin, Yu-Peng Wu, Ting-Ting Lin, Dong-Ning Chen, Yong Wei, Xue-Yi Xue, Ning Xu

**Affiliations:** Department of Urology, First Affiliated Hospital of Fujian Medical University, 20 Chazhong Road, Fuzhou, 350005 China

**Keywords:** Penoscrotal edema, Recurrent, Lower extremities, Lymphatic reflux

## Abstract

**Background:**

Penoscrotal edema is typically caused by lymphatic obstruction, which can have both primary and secondary causes. Studies describing congenital penoscrotal edema are rare. Surgery can be divided into two types: The first approach involves extensive removal of diseased tissue and tissue reconstruction. The second approach is removal of the lesions and creating additional lymphatic vascular anastomoses.

**Case presentation:**

We present a case report of a 15-year-old patient with recurrent penoscrotal edema and swelling of both lower extremities. The literature were also reviewed to provide additional information. Physical examination revealed slow lymphatic reflux of the lower extremities and no obvious abnormalities in testicular morphology, bilaterally, or blood supply. Surgery was performed by excising the affected skin and subcutaneous tissue and the flaps was cut in the middle in Y shape to cover the penis and scrotum. Postoperative follow-up revealed wound integrity and patient satisfaction with the outcome.

**Conclusion:**

Excision and reconstructive surgery are the primary treatments for penoscrotal edema. The majority of reported patients undergoing excision and reconstruction achieved satisfactory reshaping and improved their life quality.

**Electronic supplementary material:**

The online version of this article (10.1186/s12894-019-0456-6) contains supplementary material, which is available to authorized users.

## Background

Penoscrotal edema is typically caused by lymphatic obstruction, which can have both primary and secondary causes. Lymphatic malformations result from congenital causes (primary lymphedema). Acquired (secondary) chronic genital lymphedema can be caused by genital infection, tumors, lymphadenectomy, injury, or irradiation. Scrotal lymphadenopathy may be transient or persistent, and occurs at any age. For some patients, conservative treatment is sufficient and in others, the appearance of the edema or a patient’s quality of life requires surgical treatment to improve [1, 2].

Studies describing congenital penoscrotal edema are rare [3–5]. Treatment is surgical and can involve preoperative removal of the cause, as in active cases of filariasis. Penile scrotal skin with acute inflammation or ulcers must be treated preoperatively to avoid postoperative recurrence. Surgical resection of hyperplastic tissue and restoring the appearance of the penis and scrotum and sexual function are necessary to maintain the physiological function of the testes [6]. Thorough removal of the lesion produces the best results, and is most likely to reduce or eliminate recurrence [7, 8].

Surgery can be divided into two types: The first approach involves extensive removal of diseased tissue and tissue reconstruction. The second approach is removal of the lesions and creating additional lymphatic vascular anastomoses. The former is the classic approach, which is also more extensive [9]. Many techniques have been described to treat penile and scrotal elephantiasis, which differ in incision lines and covering techniques [10]. Other skin parts may be of use like posterior scrotal flaps, superiorly based flap of the pubic area for testicular coverage, and split-skin graft to the penis. Regardless which technique is used, the possibility of postoperative recurrence requires long-term follow-up.

## Case presentation

**Fig. 1:**
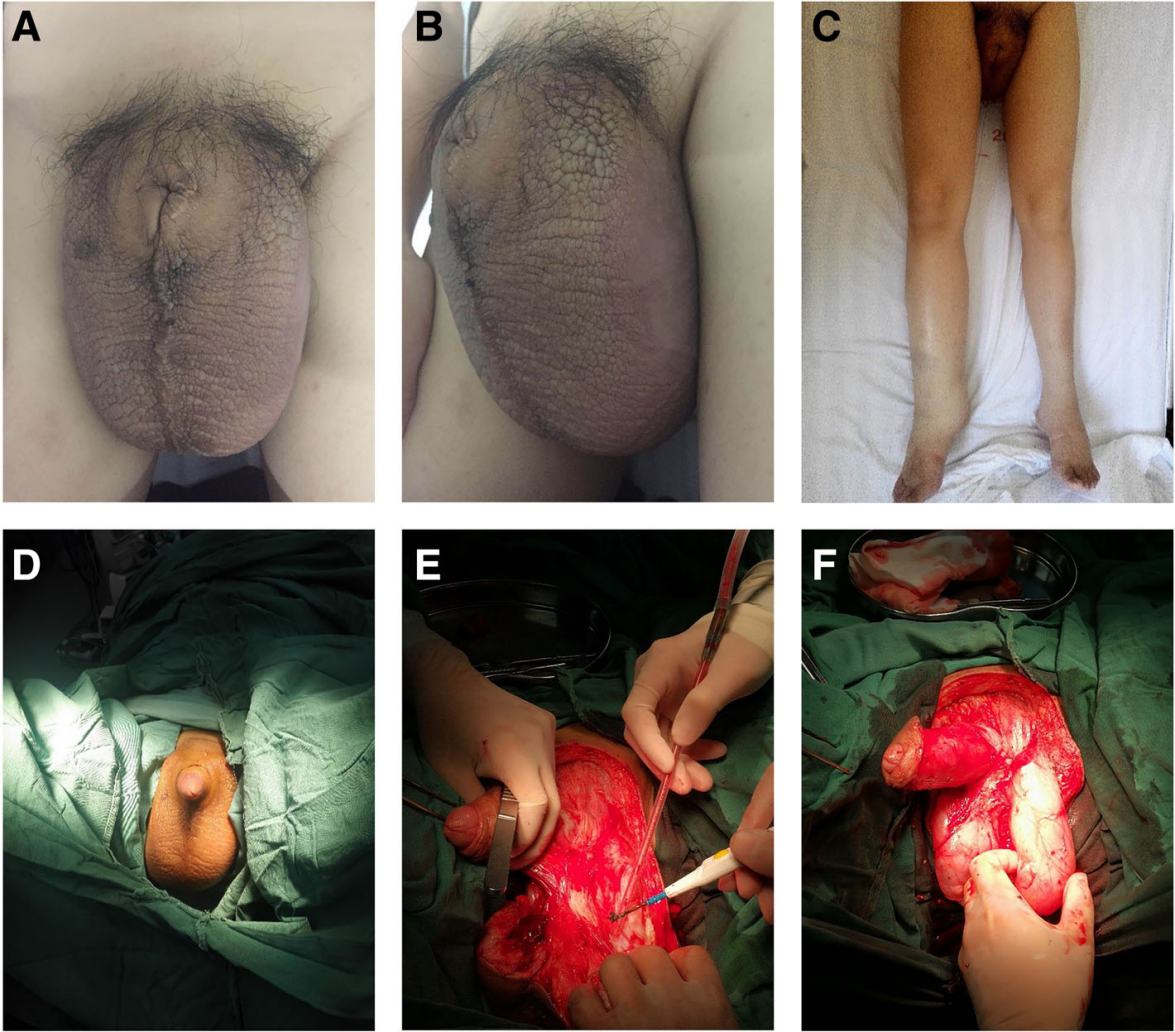
**a** Frontal view of the patient’s scrotum. **b** Side view of the patient’s scrotum. **c** Appearance of the patient’s lower extremities. **d** Surgical incision pattern. **e** and **f** Excising the skin and subcutaneous tissues

A 15-year-old boy presented to Fujian Medical University with giant scrotal elephantiasis and swelling of both lower extremities. The penoscrotal edema began fifteen years earlier, soon after his birth, and it resulted in bilateral lower extremity edema with the penis becoming buried by the scrotum. His scrotal size was massive, and for the past 5 years, the glans penis was not visible nor palpable (Fig. 1a–c). He had undergone circumcision 13 year earlier and had no history of travel in filariasis-endemic areas. There was also no family history of scrotal elephantiasis or known genetic disorders.

Upon examination, the patient had a massively enlarged scrotum, with a volume of approximately 16 cm × 13 cm × 7 cm. The anatomical structures and urethral orifice were visible as a deep depression on the scrotum. Both lower extremities exhibited generalized swelling, which was especially noticeable on his ankles. His thigh circumference was 52 cm on the left and 56 cm on the right.

**Fig. 2:**
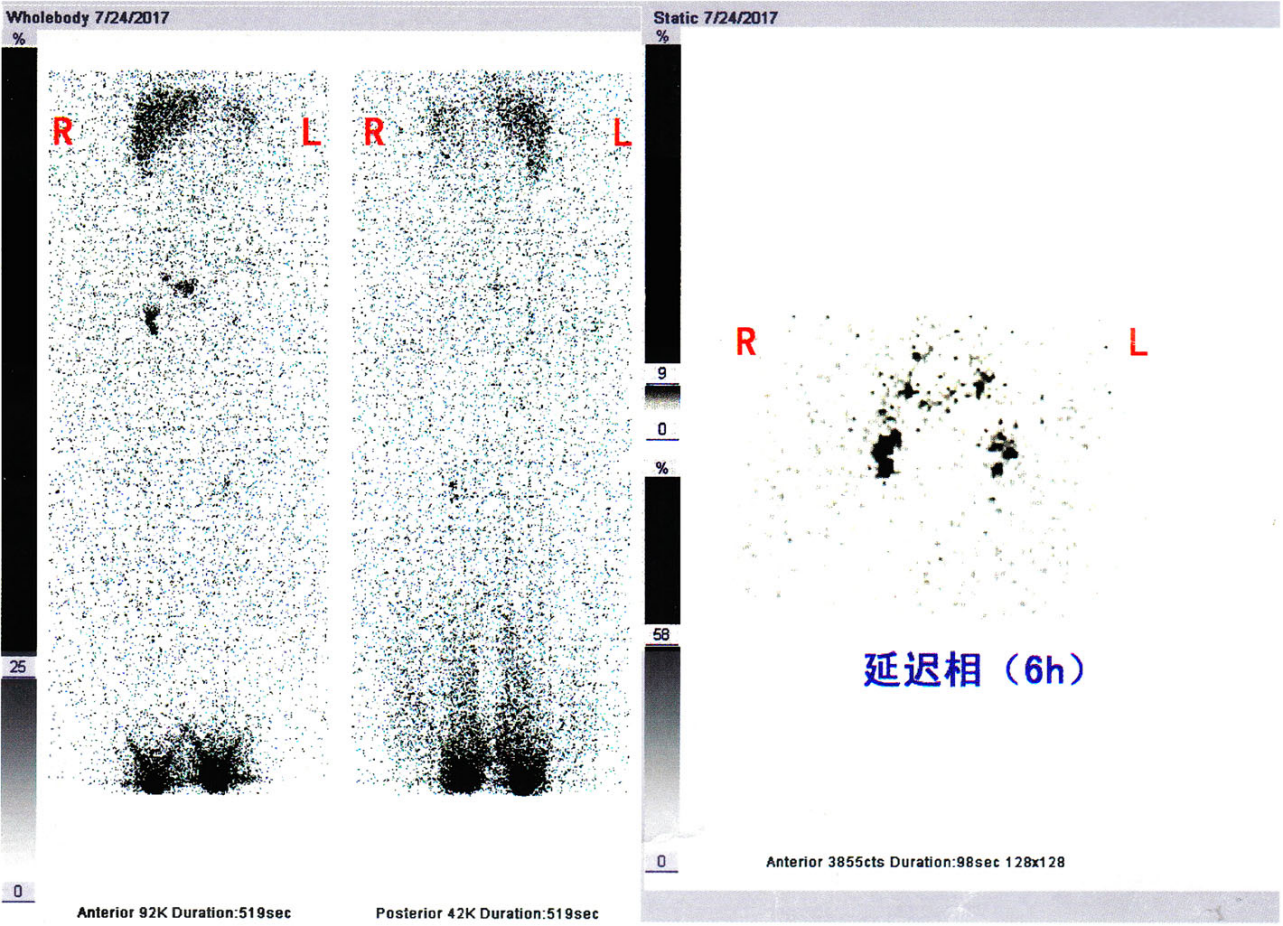
The results of the scan of lymphoscintigraphy. The lymphoscintigraphy results demonstrated that the lymphatic drainage of the lower extremities was obviously tardy. The development of bilateral inguinal and iliac lymph nodes was obviously tardy

Tissue biopsy of the lower extremities was performed 13 years earlier and revealed lymphangioma and connective tissue hyperplasia. A urinary system ultrasound examination was performed 14 months prior to presentation at our hospital, which confirmed diseased subcutaneous scrotal soft tissues with no abnormalities in the bilateral testicular morphology and blood supply. The results of lower limb lymphoscintigraphy demonstrated that the lymphatic drainage of the lower extremities was obviously tardy. The development of bilateral inguinal and iliac lymph nodes was obviously tardy (Fig. 2). The lower limbs and anterior pelvic position was imaged after injecting with the tracer (99mTc-SC) subcutaneously between the first and second toes. The images demonstrated that the lymphatic drainage in both lower extremities were unclear. In the early stage, the images showed that the bilateral inguinal and iliac lymph nodes were blurred, which was obvious on the left side. The concentration of imaging agent (99mTc-SC) in the bilateral inguinal and iliac lymph nodes was gradually increased within 6 h after imaging. There was no significant concentration of imaging agent (99mTc-SC) on the skin of the scrotum during the entire process (Additional files 1, 2, 3, and 4). Results of laboratory testing, including human immunodeficiency virus and routine blood evaluation, including a full biochemical profile, were all within the normal ranges.

**Fig. 3:**
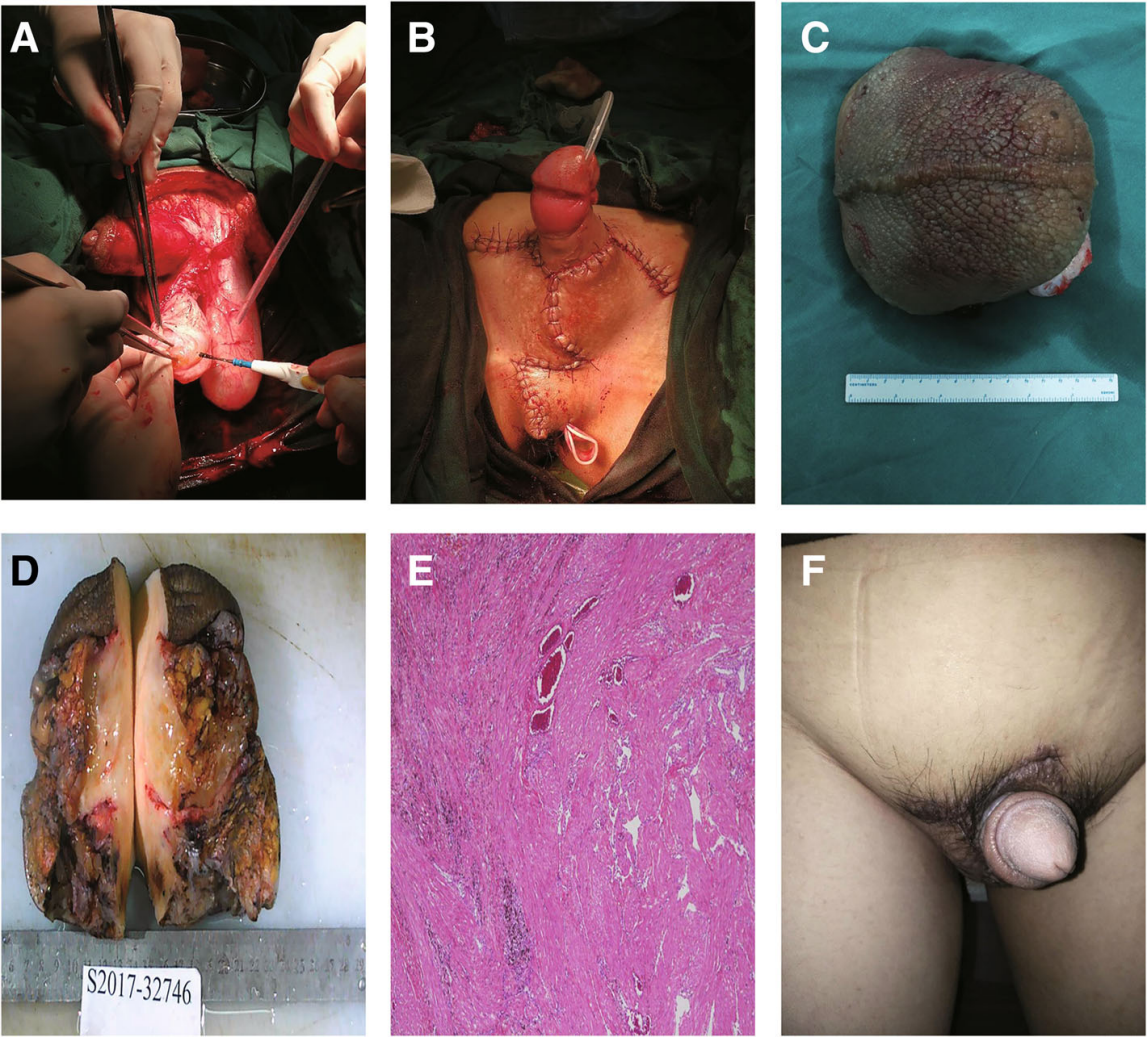
**a** Incising the testicular sheath. **b** “Y”-suture pattern to close the flaps. **c** Resected patient’s scrotum measuring 16 cm × 13 cm × 7 cm. **d** The resected scrotum with the skin removed. **e** Photomicrograph showing the histological findings in the scrotal tissue (Hematoxylin & eosin, × 100). **f** The patient’s scrotum and penis 3 months postoperatively

Surgery was performed on September 18th, 2017. The affected skin and subcutaneous tissues were excised and the flaps was cut in the middle in Y shape to cover the penis and scrotum. The primary goal of surgery was to completely remove the affected tissues. The incision began at the side of the groin outside the outer ring then ran underneath the scrotum and sagittally forward and downward, then back towards the rear of the scrotum, where it then ran around the back of the scrotum. The incision was on the midline, with the contralateral incision rendezvous point in front of the incision from the top to the top of the extension, which was at the midline near the root of the penis and the join with the contralateral incision (Fig. 1d). The skin was freed on both sides of the flap to the scrotum on the outside, the thickened scrotal wall was transected, and the scrotal lesions were removed (Fig. 1e and f). Bilateral testicular hydroceles were found intraoperatively that measured approximately 6.0 cm × 6.0 cm × 5.0 cm. Therefore, the testicular sheath was incised, which released thick brown fluid; the cavity of the tunica vaginalis had no connection with the abdominal cavity (Fig. 3a). We then sutured the flaps with a “Y” suture on both sides to reconstruct the scrotum, placing a drain distally (Fig. 3b). To address the swollen extremities, we adopted conservative treatment, such as raising both lower limbs and wearing elastic stockings to improve lymphatic reflux.

The excised scrotal tissue weighed 5.2 kg (Fig. 3c and d). Histopathological examination did not reveal the presence of microorganism or parasites, and confirmed lymphangia with fibroblast proliferation and previous hemorrhage (Fig. 3e). Three months postoperatively, his scrotal appearance and penile function had improved (Fig. 3f), with evident wound integrity and patient satisfaction with the outcome (Fig. 1b).

Important technical points in this surgical treatment include the complete dissection of all involved tissue, and using scrotal advancement flaps from areas with normal, non-edematous skin. Other skin parts may be of use like posterior scrotal flaps, superiorly based flap of the pubic area for testicular coverage, and split-skin graft to the penis. This case shows that surgical therapy can provide good functional and cosmetic results in scrotal elephantiasis.

## Discussion and conclusions

Penoscrotal edema is a condition of localized fluid retention and tissue swelling caused by a compromised lymphatic system. The condition may be inherited (primary) or caused by obstruction or disruption in the lymphatic vessels (secondary), which develops more frequently than primary lymphedema [1, 2, 11]. The etiologies of secondary penoscrotal edema include nodal dissection, neoplastic disease, surgery, injury, radiation, rheumatoid arthritis, filariasis, recurrent infection, and idiopathic causes [12].

Patients most often suffer from a sense of heaviness and fatigue. In later stages, the hyperkeratotic skin forms a vesicle filled with exudative lymph. The damaged skin barrier allows bacteria into the protein-rich lymphoid fluid in the vesicle, which frequently leads to cellulitis and postoperative complications such as abscesses, wound infections, and wound dehiscence.

The main purpose of surgery is to reduce scrotal volume, reconstruct the scrotum, and repair the skin of the penis [10]. A previous report discussed the treatment of 48 cases of penile and penile/scrotal filariasis-related lymphedema using plastic surgery. The scrotal skin and related soft tissue were removed in all patients followed by scrotal plastic surgery, while retaining the testes and spermatic cord. All 48 patients achieved satisfactory reshaping after surgery, were able to walk better, and sexual function was restored [7].

Cases of penoscrotal edema are rare, and some reported cases are summarized in Table 1. All reported patients to date were adults aged 22–65 years, with no history of surgery, irradiation, or travel to filariasis-endemic regions, as in our patient. Our patient, a 15-year-old boy, suffered penoscrotal edema soon after his birth, which is much younger than in other reports. The reported scrotal weight in cases of penoscrotal edema ranged from 1.45–40.5 kg.

**Table 1:** Descriptive averaged statistics of reported cases of penoscrotal edema

First author/s (ref)	Age	Time of Penoscrotal edema	History	Weigh of penoscrotal edema/kg	Dimension (cm)	Treatment	Histologic examination	Follow up time, year	Recurrence
Evangelos Zacharakis [14]	65	6	NO	1.45	15*10	Excision and reconstruction surgery	smooth muscle hyperplasia, lymphatic vessels	3	NO
Badrinath R. Konety [13]	46	5	NO	11.3	60 *50 *30	Excision and reconstruction surgery	extensive fibrosis, microabsceses, hidardenitis suppurativa and chronic lymphedema	1/4	NO
Zekeriya Tosun [15]	65	2	NO	40.5	65* 50* 20	Excision and reconstruction surgery	chronic inflammation	2	NO
GONG-KANG HUANG [16]	31	15	NR	NR	16.5*20	Microlymphaticovenous anastomosis	NR	2	NO
GONG-KANG HUANG [16]	65	3	NR	NR	12* 13.5	Microlymphaticovenous anastomosis	NR	1.5	NO
GONG-KANG HUANG [16]	53	6	NR	NR	11* 18	Microlymphaticovenous anastomosis	NR	1.25	NO
EDGAR D. [17]	32	4	NR	NR	22.8 cross	Excision and reconstruction surgery	NR	2	NO
PERRY BONAR [18]	40	NR	NO	NR	50.8*35.5	Excision and reconstruction surgery	connective tissue proliferation, interstitial edema and lymphatic vessels.	2/3	NO
Badr Alharbi [19]	38	5	NR	NR	NR	Excision and reconstruction surgery	NR	NR	NR
Manish C [8]	52	5	NR	NR	24.0*15.0* 6.0	Excision and reconstruction surgery	dermal fibrosis, perivascular chronic inflammation	1	NO
Hilary Laurel Brotherhood [20]	65	NR	NO	NR	NR	Excision and reconstruction surgery	NR	1	NO
Hilary Laurel Brotherhood [20]	43	10	NO	2	NR	Excision and reconstruction surgery	NR	3/4	NO
DIANZANP [21]	37	6	NO	NR	50* 47* 13	Excision and reconstruction surgery	non-specific chronic inflammation	1	NO
J Wiblin [22]	40	24	NO	NR	40.6* 33.0	Excision and reconstruction surgery	fibro-cellular tissue	NR	NR
Stefan Denzinger [23]	40	10	NO	NR	65* 55* 25	Excision and reconstruction surgery	Non-specific chronic inflammation	NR	NR
D Kuepper [5]	45	9	NR	42	80* 40*40	Excision and reconstruction surgery	NR	NR	NR
Poornachandra Thejeswi [24]	54	8	NR	32	NR	Excision and reconstruction surgery	extensive fibrosis tissue and lymphostasis	NR	NR
BRAD J. HORNBERGER [25]	22	5	NO	11.3	33.5* 20.6	Excision and reconstruction surgery	smooth muscle hyperplasia, dermal fibrosis and chronic inflammation	NR	NR

Author is the first author’s first and last name; Age is averaged patient’s years of age; History is history of surgery, irradiation, or travel to filariasis-endemic regions; NR is not recorded

Pathological results have not been reported in every previous study. However, the most significant microscopic features in previous reports were non-specific inflammation with connective tissue proliferation; hidradenitis suppurativa was found in Konety et al.’s study [13]. The penoscrotal edema in our patient was pathologically confirmed as lymphangioma with fibroblast proliferation and previous hemorrhage, with no specific cause.

Excision and reconstructive surgery are the primary treatments for penoscrotal edema. The majority of reported patients undergoing excision and reconstruction achieved satisfactory reshaping and improved their life quality to some degree within a 2–3-year follow-up, including patients who underwent microlymphaticovenous anastomosis. No recurrence was reported in the previous reports listed in our study. Our patient’s incisions healed in 2 weeks, with good final appearance and satisfactory erectile function by 3 months, postoperatively.

## Additional files

Additional file 1: Images of the scan of lymphoscintigraphy (Transverse section). (JPG 1114 kb)

Additional file 2: Images of the scan of lymphoscintigraphy (Transverse section). (JPG 1118 kb)

Additional file 3: Images of the scan of lymphoscintigraphy (Coronal section). (JPG 1112 kb)

Additional file 4: Images of the scan of lymphoscintigraphy (Three-dimensional reconstruction). (JPG 896 kb)
