# Supplementary figures and images for: Penoscrotal edema: a case report and literature review
Source: BMC Urol. 2019 Apr 15;19:22. doi: 10.1186/s12894-019-0456-6 (PMC6466797; doi:10.1186/s12894-019-0456-6)

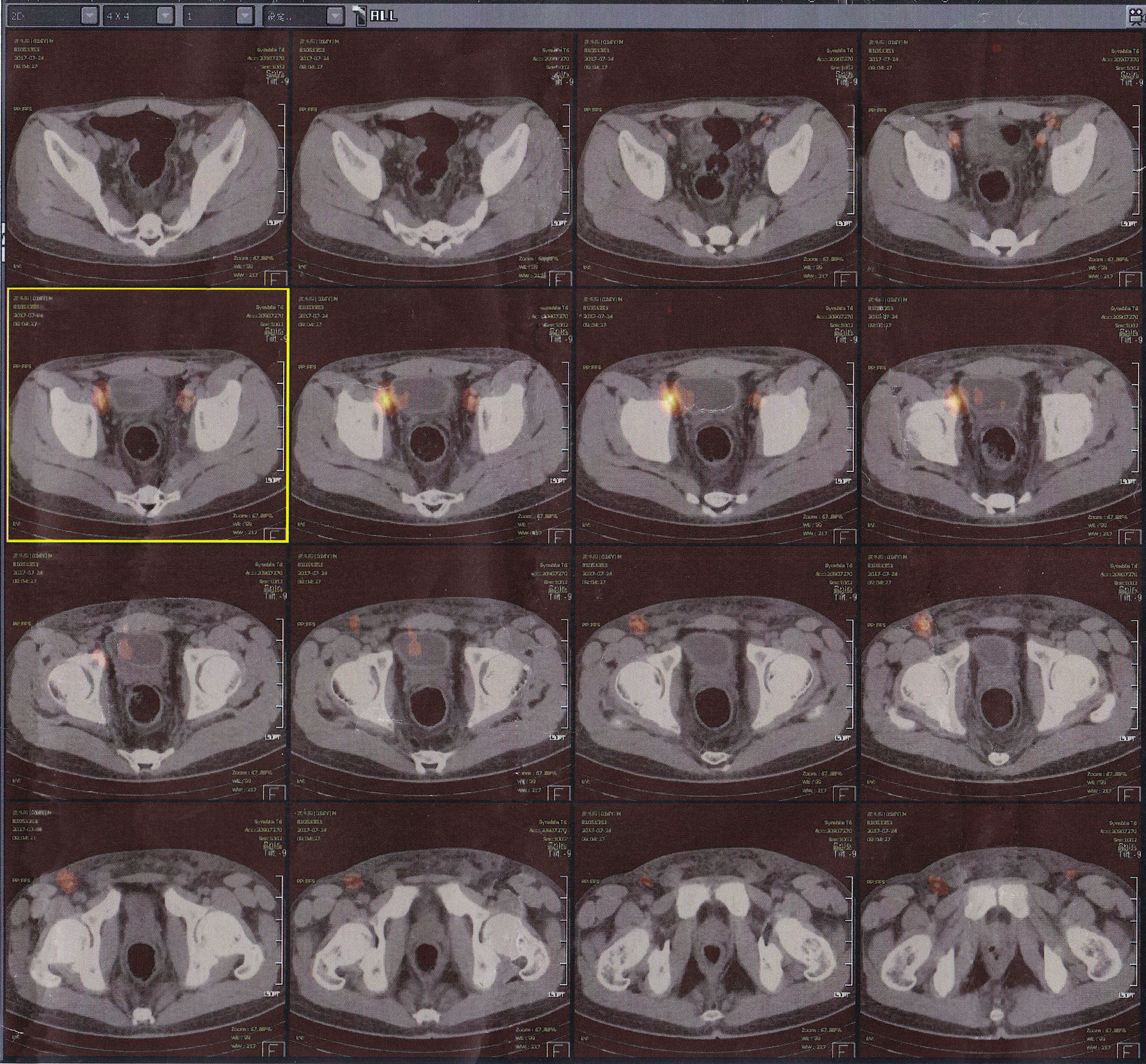

Supplement: Supplementary file 1 — Images of the scan of lymphoscintigraphy (Transverse section). (JPG 1114 kb) [file 12894_2019_456_MOESM1_ESM.jpg]

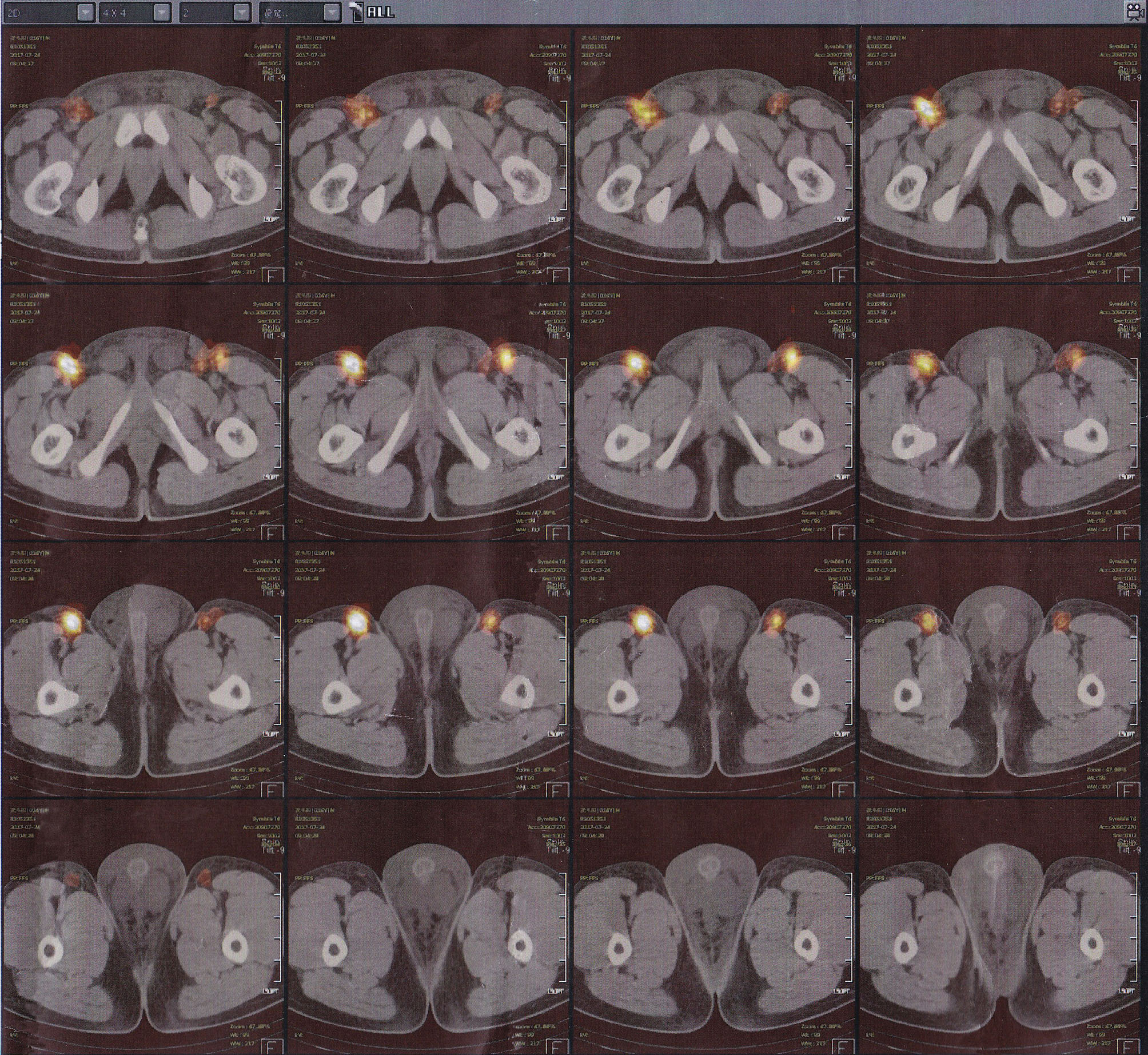

Supplement: Supplementary file 2 — Images of the scan of lymphoscintigraphy (Transverse section). (JPG 1118 kb) [file 12894_2019_456_MOESM2_ESM.jpg]

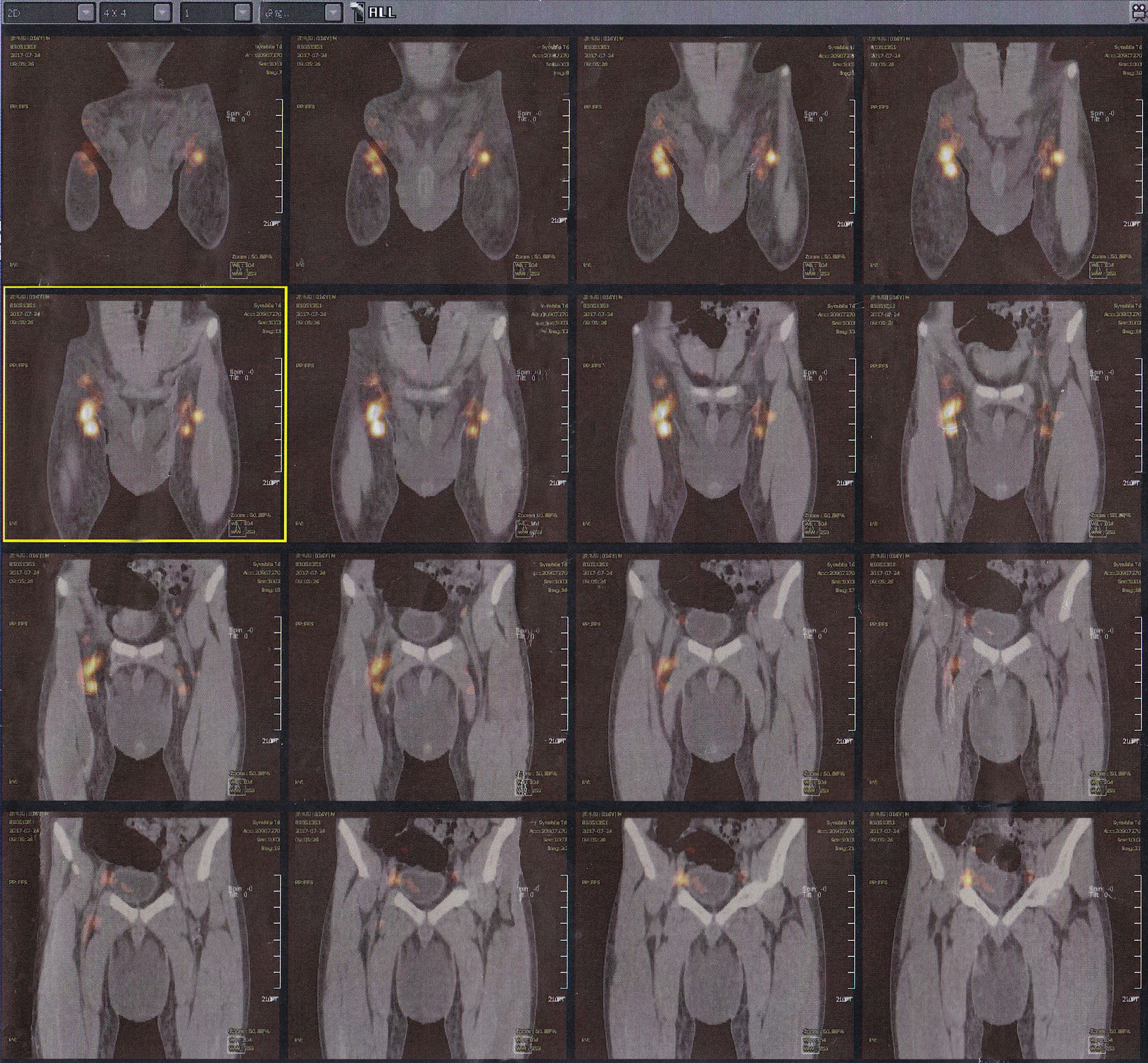

Supplement: Supplementary file 3 — Images of the scan of lymphoscintigraphy (Coronal section). (JPG 1112 kb) [file 12894_2019_456_MOESM3_ESM.jpg]

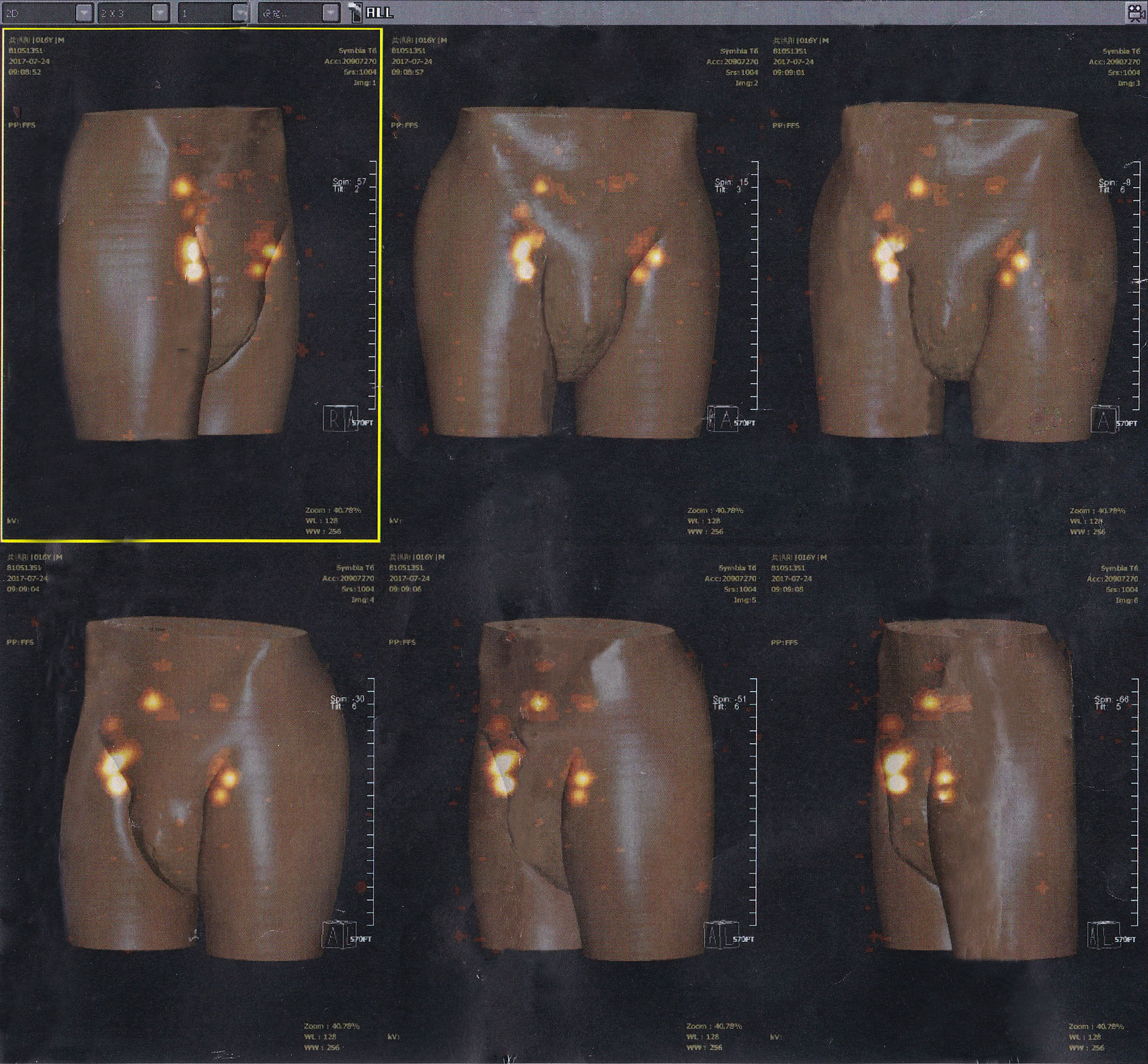

Supplement: Supplementary file 4 — Images of the scan of lymphoscintigraphy (Three-dimensional reconstruction). (JPG 896 kb) [file 12894_2019_456_MOESM4_ESM.jpg]
